# A Lipoprotein Lipase–Promoting Agent, NO-1886, Improves Glucose and Lipid Metabolism in High Fat, High Sucrose–Fed New Zealand White Rabbits

**DOI:** 10.1080/15438600303732

**Published:** 2003

**Authors:** Weidong Yin, Zhonghua Yuan, Kazuhiko Tsutsumi, Yuxiang Xie, Qiuju Zhang, Zongbao Wang, Guoxiang fu, Guang Long, Yongzong Yang

**Affiliations:** 1 Department of Pathophysiology Central South University Xiangya Medical College Changsha China; 2 Institute of Cardiovascular Research Nanhua University Medical College Hengyang Hunan China; 3 Research and Development Otsuka Pharmaceutical Factory Inc. Tokushima Japan; 4 Department of Biochemistry and Molecular Biology Nanhua University Medical College Hengyang Hunan 421001 China

## Abstract

The synthetic compound NO-1886 is a lipoprotein lipase activator that lowers plasma triglycerides and elevates high-density lipoprotein cholesterol (HDL-C). Recently, the authors found that NO-1886 also had an action of reducing plasma glucose in high-fat/high-sucrose diet–induced diabetic rabbits. In the current study, we investigated the effects of NO-1886 on insulin resistance and *β*-cell function in rabbits. Our results showed that high-fat/high-sucrose feeding increased plasma triglyceride, free fatty acid (FFA), and glucose levels and decreased HDL-C level. This diet also induced insulin resistance and impairment of acute insulin response to glucose loading. Supplementing 1% NO-1886 into the high-fat/high-sucrose diet resulted in decreased plasma triglyceride, FFA, and glucose levels and increased HDL-C level. The authors also found a clear increased glucose clearance and a protected acute insulin response to intravenous glucose loading by NO-1886 supplementation. These data suggest that NO-1886 suppresses the elevation of blood glucose in rabbits induced by feeding a high-fat/high-sucrose diet, probably through controlling lipid metabolism and improving insulin resistance.


*Experimental Diab. Res.*, 4:27–34, 2003
Copyright © 2003 Taylor & Francis
1543-8600/03 $12.00 + .00
DOI: 10.1080/15438600390214608

Taylor & Francis healthsciences

### A Lipoprotein Lipase–Promoting Agent, NO-1886, Improves Glucose and Lipid Metabolism in High Fat, High Sucrose–Fed New Zealand White Rabbits

**Weidong Yin,[1,4] Zhonghua Yuan,[2] Kazuhiko Tsutsumi,[3] Yuxiang Xie,[2] Qiuju Zhang,[2] Zongbao Wang,[2] Guoxiang fu,[2] Guang Long,[2] and Yongzong Yang[1,2]**

[1]*Department of Pathophysiology, Central South University Xiangya Medical College, Changsha, P. R. China*
[2]*Institute of Cardiovascular Research, Nanhua University Medical College, Hengyang, Hunan, P. R. China*
[3]*Research and Development, Otsuka Pharmaceutical Factory Inc., Tokushima, Japan*
[4]*Department of Biochemistry and Molecular Biology, Nanhua University Medical College, Hengyang, Hunan, P. R. China*

**The synthetic compound NO-1886 is a lipoprotein lipase activator that lowers plasma triglycerides and elevates high-density lipoprotein cholesterol (HDL-C). Recently, the authors found that NO-1886 also had an action of reducing plasma glucose in high-fat/high-sucrose diet–induced diabetic rabbits. In the current study, we investigated the effects of NO-1886 on insulin resistance and $\beta$-cell function in rabbits. Our results showed that high-fat/high-sucrose feeding increased plasma triglyceride, free fatty acid (FFA), and glucose levels and decreased HDL-C level. This diet also induced insulin resistance and impairment of acute insulin response to glucose loading. Supplementing 1% NO-1886 into the high-fat/high-sucrose diet resulted in decreased plasma triglyceride, FFA, and glucose levels and increased HDL-C level. The authors also found a clear increased glucose clearance and a protected acute insulin response to intravenous glucose loading by NO-1886 supplementation. These data suggest that NO-1886 suppresses the elevation of blood glucose in rabbits induced by feeding a high-fat/high-sucrose diet, probably through controlling lipid metabolism and improving insulin resistance.**

**Keywords** CAS 133208-93-2; Diabetes; Free Fatty Acids; Glucose; Insulin Resistance; NO-1886; Rabbits; Triglycerides

Chronic hyperglycemia and hyperlipidemia can exert deleterious effects on $\beta$-cell function, respectively referred to as glucotoxicity and lipotoxicity. Over time, both contribute to the progressive deterioration of glucose homeostasis characteristic of type 2 diabetes [1–5]. Bearing the glucotoxicity and lipotoxicity theories in mind, we recently established a high-fat/high-sucrose diet–induced rabbit diabetes model, which is useful for the studies of glucose and lipid metabolism [6, 7].

NO-1886, a lipoprotein lipase–promoting agent, was primarily developed as a hypolipidemic agent. Administration of NO-1886 to experimental animals increased the postheparin plasma low-density lipoprotein (LPL) activity and resulted in a reduction in plasma triglyceride (TG) concentration with a concomitant increase in high-density lipoprotein cholesterol (HDL-C) concentration [8–14]. Significant elevations in plasma TG and very-low-density lipoprotein cholesterol

Received 28 August 2002; accepted 11 January 2003.
Address correspondence to Weidong Yin, MD, PhD, Professor, Department of Biochemistry and Molecular Biology, Nanhua University Medical College, Hengyang, Hunan 421001, P. R. China. E-mail: wdy20012001@yahoo.com

(VLDL-C), and significant reduction in HDL-C, appear to be characteristic in diabetic patients [15–18]. Treatment of diabetes by low-energy diets, oral hypoglycemic agents, and insulin therapy frequently is not sufficient for perfect control of lipid metabolism [16]. So, it is very important to develop drugs that have potential effects on diabetic dyslipidemia. According to the lipotoxicity theory, a drug, such as NO-1886, that has a potential effect on hypertriglyceridemia may have beneficial actions in glucose metabolism. Recently, we did find that NO-1886 also had an action of reducing plasma glucose in high-fat/high-sucrose diet–induced rabbit diabetes [7]. However, the mechanisms by which NO-1886 reduces plasma glucose cannot be clearly explained from the results of our previous study without performing the glucose tolerance test or insulin tolerance test in the animals. Therefore, in the current study, we investigated the effects of NO-1886 on glucose, insulin, and lipid profiles in high fat/high sucrose–induced mild diabetic New Zealand White rabbits.

### MATERIALS AND METHODS

### Materials

Agent NO-1886 ([4-(4-Bromo-2-cyano-phenylcarbamoyl)-benzyl]-phophonic acid diethyl ester; CAS 133208-93-2, Lot. No. 1A77M) was synthesized in the New Drug Research Laboratory of Otsuka Pharmaceutical Factory Inc. (Tokushima, Japan). Sucrose was obtained from Liuzhou Sugar Company (Guangxi, China) and lard was obtained from Hengyang Meat Product Company (Hunan, China). All other chemicals used were high-grade commercially available products.

### Animal and Diets

All animal experiments were approved by the local animal ethics committee of Nanhua University Medical College. Male New Zealand White rabbits, weighing approximately 2 kg, were obtained from the Sheng Wan Experimental Animal Ranch (Shanghai, China). The animals were randomly assigned into 4 groups: (A) normal group, which received regular rabbit chow (Standard Laboratory Chow, Sheng Wan Experimental Animal Ranch, Shanghai, China) for 28 weeks; (B) normal NO-1886 group, which was fed regular rabbit chow for the first 4 weeks, then supplemented with 1.0% NO-1886 for the remaining 24 weeks; (C) sucrose group, which was fed high-fat/high- sucrose chow (incorporating 10% lard and 37% sucrose into the standard laboratory chow, prepared in our institute [6]) for 28 weeks; (D) sucrose NO-1886 group, which was fed high-fat/high-sucrose chow for the first 4 weeks, then supplemented with 1.0% NO-1886 for the remaining 24 weeks. Every group had 12 rabbits. The rabbits were maintained in a 12-hour light-dark cycle at a constant temperature of 23°C ± 2°C. The animals were fed at 9 AM, given an amount of 35 g/kg/day, and given free access to tap water. Food consumption was measured daily and the treated rabbits were trained to consume completely the whole allotted portion of the food containing NO-1886 within an hour. At the end of the experimental period, the animals were killed by exsanguinations under sodium pentobarbital anesthesia. The abdominal fat was removed and weighed.

### Metabolic Measurements

Body weights were measured at the time points as indicated. Blood samples for fasting glucose, insulin, and lipid measurements were withdrawn from auricular veins at weeks 0, 4, 8, 13, 18, 23, and 28 after fasting overnight, kept at room temperature for 30 minutes, and then centrifuged for 10 minutes, at 500 × *g* (Beckman GPR, Palo Alto, CA). The serum was then collected and frozen at −20°C until assayed. Glucose tolerance test was performed on conscious animals following an overnight fast by intravenous administration of glucose (2 g/Kg). The blood samples for measurements of glucose and insulin were withdrawn at 0, 5, 15, 30, 60, and 120 minutes. The insulin tolerance test was done on fed conscious rabbits. Human recombinant insulin (Shanghai Biochemistry factory, Shanghai, China; 0.75 units/kg body weight) was injected intraperitoneally to the animals after appropriate dilution with saline. The blood samples were withdrawn at 0, 15, 30, and 60 minutes after insulin administration served for determination of glucose.

### Analytical Methods

Serum HDL-C (HDL-C Test Kit, Shanghai Rongsheng Biotech Inc.), triglycerides (TG Test Kit, Shanghai Rongsheng Biotech Inc.), free fatty acids (FFA Test Kit, Shanghai Rongsheng Biotech Inc.), and glucose (glucose oxidase–peroxidase method, Shanghai Rongsheng Biotech Inc.) were determined by commercially enzymatic methods. Insulin was determined by conventional radioimmunoassay, with the use of Insulin Radioimmunology kit (Beijing Institute of Atomic Research, Beijing, China).

### Statistical Analysis

Values are reported as mean ± SD. Comparisons between groups were analyzed for statistical significance using the 1-way analysis of variance, followed by the Dunnett's test multiple comparisons. *P* values less than .05 were considered significant.

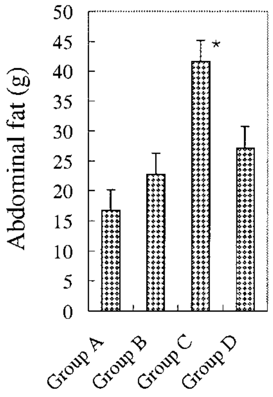

**FIGURE 1**
Accumulation of abdominal fat in the rabbits. The amount of abdominal fat was significantly higher in the rabbits of group C than in the other 3 groups. Significance: $^*P < .05$ versus group A, group B, and group D. n = 5–7.

### RESULTS

### Body Weight and Abdominal Fat

The mean body weight in animals of group C was the highest, though it was not significantly different from the other 3 groups during the experimental term of 28 weeks, but the amount of abdominal fat was significantly higher in the rabbits of group C than in the other 3 groups (Figure 1). This is a confirmation for a previous study that had shown that NO-1886 suppressed fat accumulation in rats fed a high-fat diet [11].

### Effects of NO-1886 on Lipid Metabolism

Feeding high-fat/high-sucrose diets elevated serum TG and free fatty acid (FFA) levels. However, the increase of serum TG and FFAs in group D was inhibited by supplementing 1% NO-1886 into the high-fat/high-sucrose diets (Figures 2 and 3). This action of NO-1886 on blood FFAs was a new finding.

Administration of NO-1886 significantly increased serum HDL-C levels in normal (group B) and high fat/high sucrose–fed NO-1886–supplemented (group D) rabbits (Figure 4).

### Effects of NO-1886 on Fasting Blood Glucose

Fasting blood glucose level in rabbits from group D was the highest (though it did not reach the significant level) among 4 groups by the first 4 weeks of high-fat/high-sucrose feeding. However, the increase of glucose level in group D was inhibited by supplementing 1% NO-1886 at the 8th week time point (4 weeks after start of NO-1886 administration). Fasting blood glucose level in rabbits from group D was significantly lower than in rabbits from group C at the 23rd and 28th weeks (Figure 5).

There were no significant differences in fasting serum insulin levels among the 4 groups during the experimental period (data not shown).

### Glucose Tolerance and Insulin Response to Glucose Injection

Figure 6 shows blood glucose and insulin during intravenous glucose tolerance test (IVGTT) carried out in overnight-fasted rabbits. There were no differences in blood

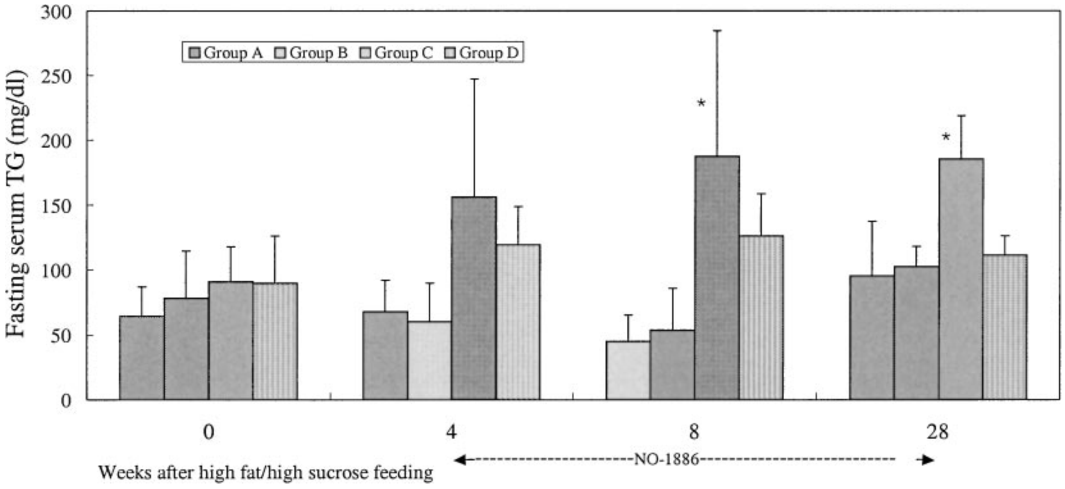

**FIGURE 2**
Time course of serum triglyceride levels after NO-1886 administration. The increase of serum triglyceride group D was inhibited by supplementing 1% NO-1886 into the high-fat/high-sucrose diets. Significance: $^*P < .05$ versus group A. n = 5–12.

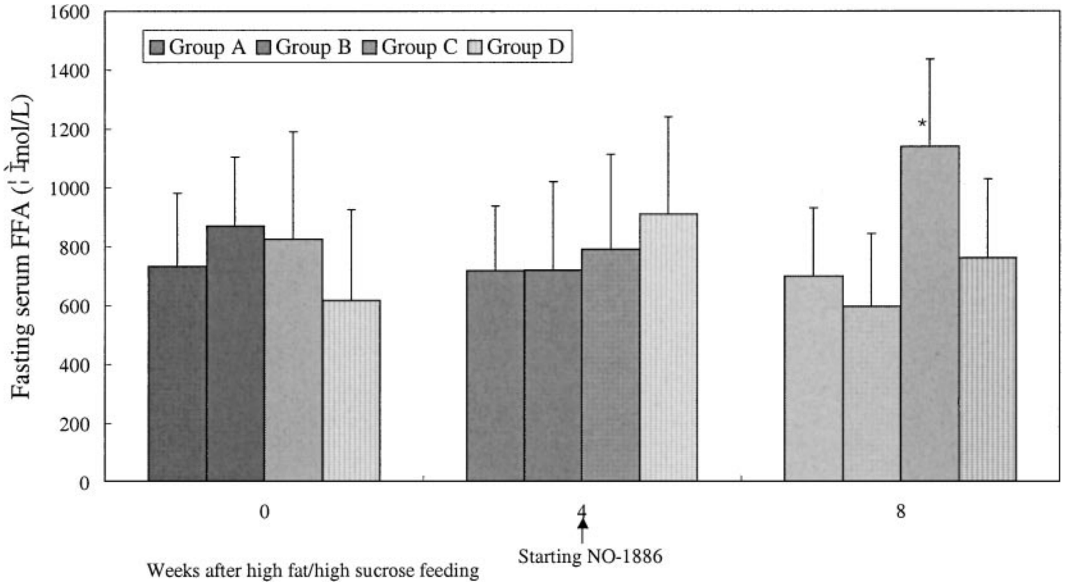

**FIGURE 3**
Effect of NO-1886 administration on serum FFA levels. Feeding high-fat/high-sucrose diets elevated plasma FFA levels. The supplementing 1% NO-1886 inhibited the increase of FFAs in group D. Significance: $^*P < .05$ versus group A. n = 10–12.

glucose (Figure 6*A*) and insulin (not shown) among 4 groups during IVGTT carried out at week 0. However, blood glucose was significantly higher in rabbits from group C versus the rabbits from the other 3 groups at all time points, except at time 5 minutes, investigated after the glucose injection at week 13 (Figure 6*B*) and at week 28 (not shown). After the intravenous glucose administration, serum insulin at time point 15 minutes (Figure 6*C*) increased to a higher extent in the group B and group D rabbits than the rabbits in group A; but in the rabbits of group C, plasma insulin at time point 15 minutes

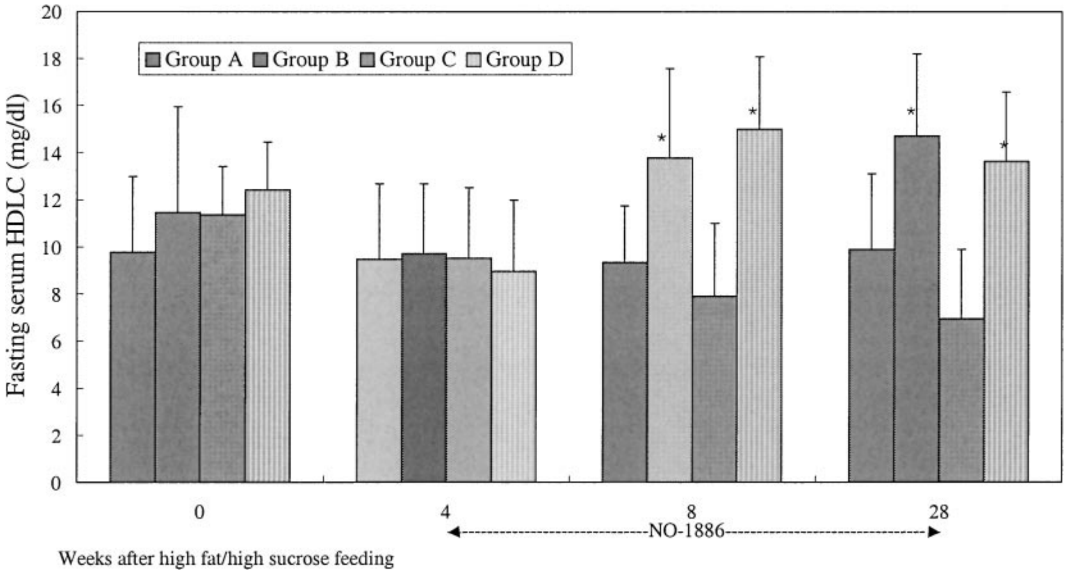

**FIGURE 4**
Time course of serum HDLc levels after NO-1886 administration. NO-1886 significantly increased serum HDL-C levels in normal (group B) and high fat/high sucrose–fed NO-1886–supplemented (group D) rabbits. Significance: $^*P < .05$ versus group A. n = 5–12.

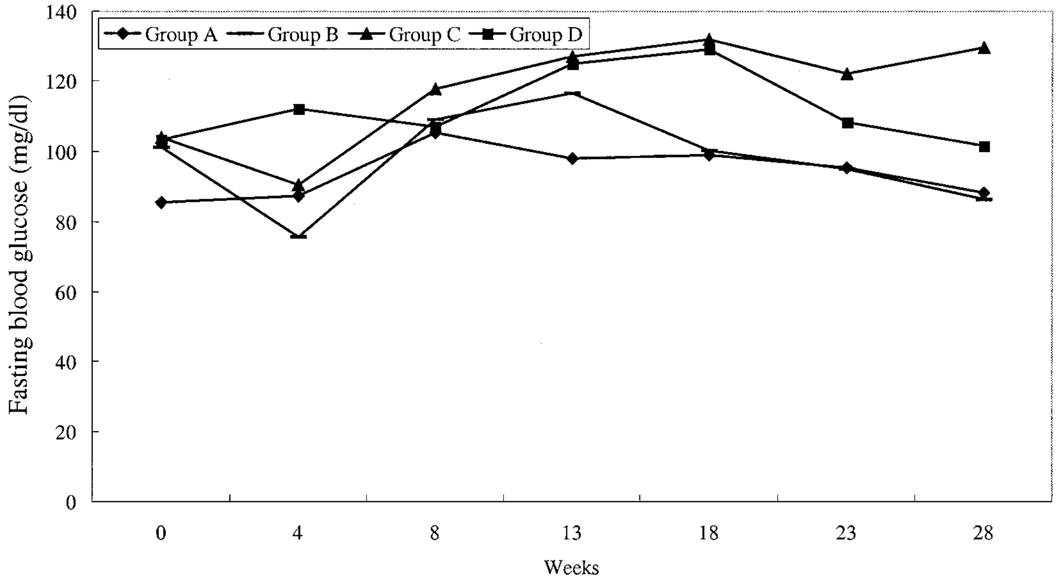

**FIGURE 5**

Time course of blood glucose levels after NO-1886 administration. The increase of glucose level induced by high-fat high-sucrose diet in group D was inhibited by supplementing 1% NO-1886 (4 weeks after start of NO-1886 administration). Fasting blood glucose level in rabbits from group D was significantly lower than in rabbits from group C at the 23rd and 28th weeks.

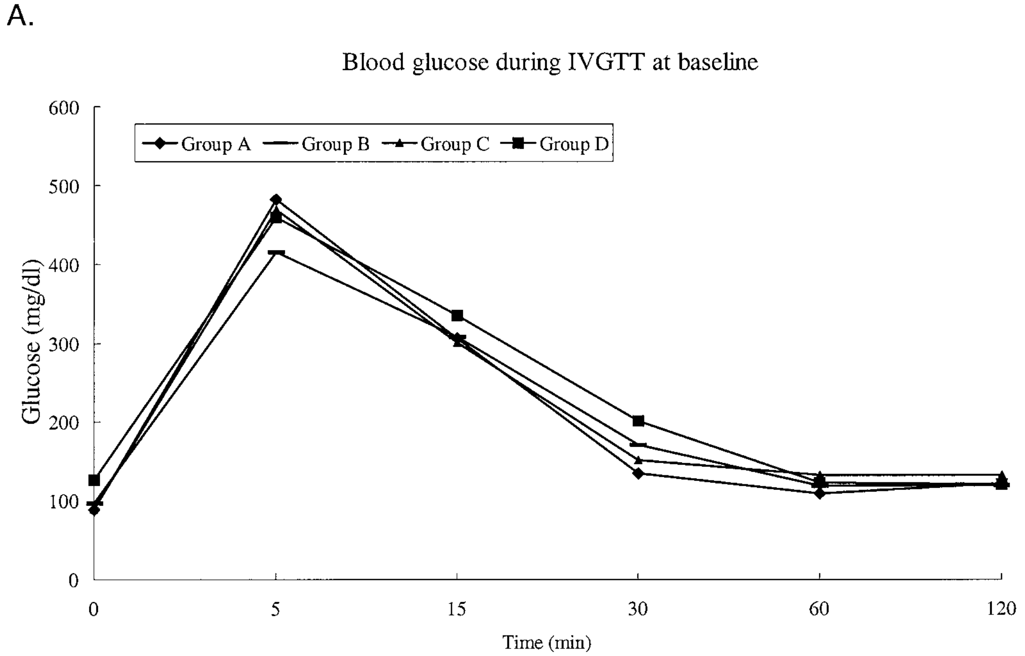

**FIGURE 6**

Serum glucose and insulin during IVGTT carried out in overnight-fasted rabbits. There were no differences in blood glucose (*A*) among the 4 groups during IVGTT carried out at week 0. However, blood glucose was significantly higher in rabbits from group C versus the rabbits from the other 3 groups at all time points, except at time 5 minutes, investigated after the glucose injection at week 13 (*B*). After the intravenous glucose administration, serum insulin at time point 15 minutes (*C*) increased to a higher extent in group B and group D, but in group C, plasma insulin at time point 15 minutes was significantly lower than the other 3 groups investigated at week 13. *(Continued)*

B.

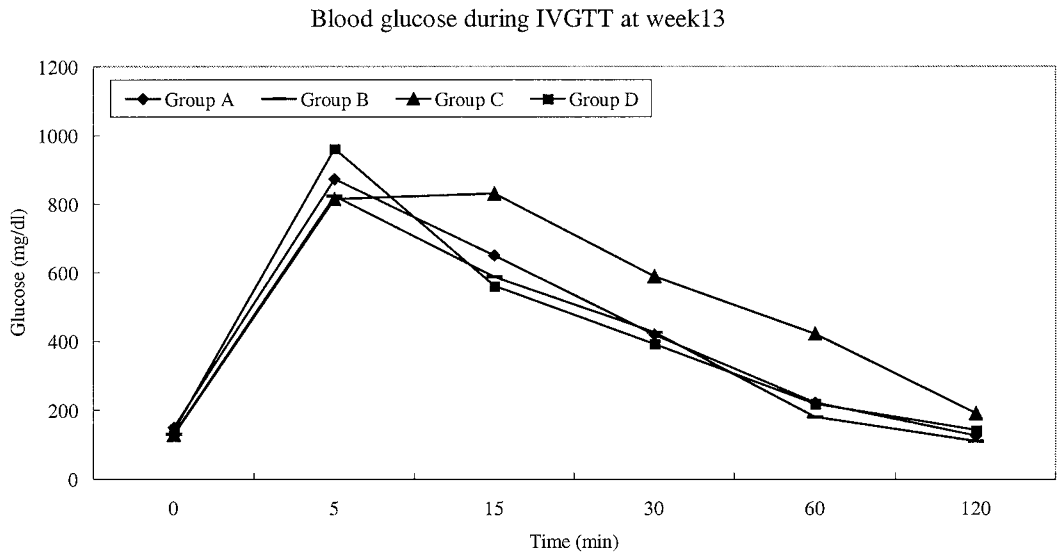

C.

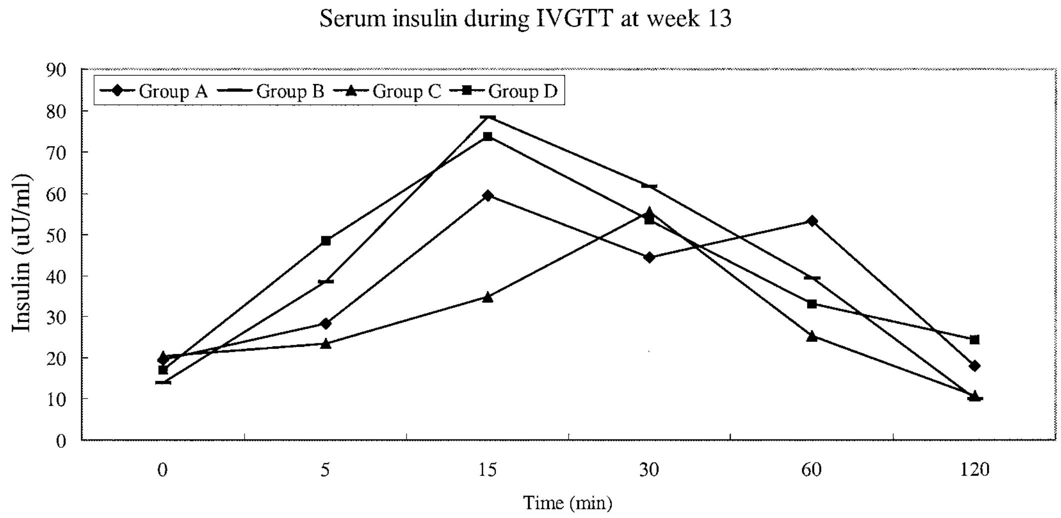

**FIGURE 6**
(Continued)

was significantly lower than the other 3 groups investigated at week 13. This indicated that high-fat/high-sucrose feeding impaired the acute insulin response to the glucose challenge in animals from group C and NO-1886 protected the acute insulin response in animals from group D. This effect of the compound was likely through reduced lipotoxicity.

### Insulin Tolerance Test

Figure 7 shows blood glucose levels after an intraperitoneal administration of insulin (0.75 units/kg body weight) in fed animals at week 13. Insulin injection caused a decrease of glucose levels with respect to basal glucose values by about 30% in group A, group B, and group D at 15, 30, and 60 minutes, whereas the decrease of blood glucose in group C was significantly lower. This suggests that NO-1886 improves insulin sensitivity in high fat/high sucrose–fed rabbits.

### DISCUSSION

It has previously been reported that NO-1886 increases LPL activity in postheparin plasma, and produces a reduction in plasma TG level with concomitant elevation of HDL-C level in animals with lipid disorder [8–13]. However, there

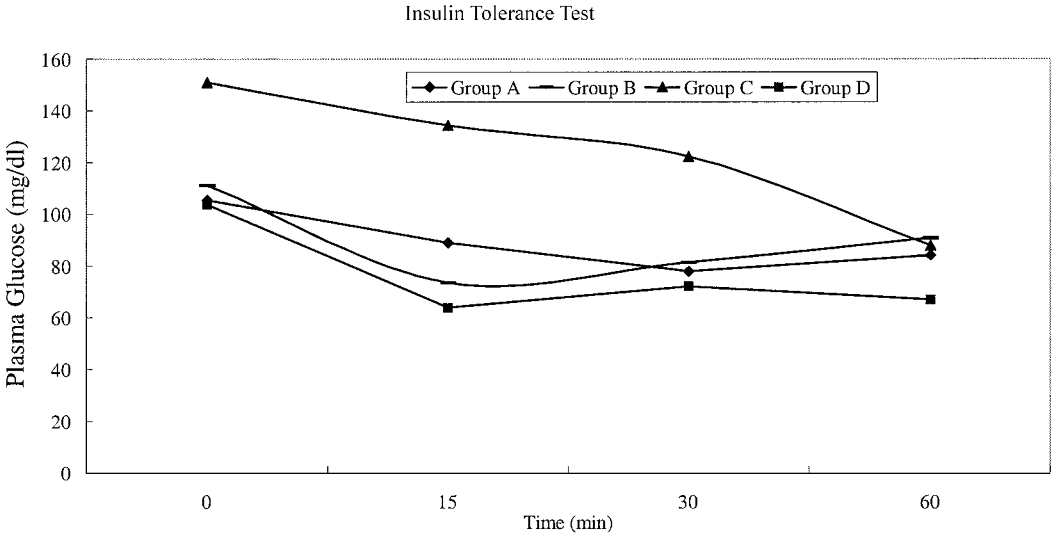

**FIGURE 7**
Serum glucose levels after an intraperitoneal administration of insulin (0.75 units/kg body wt) in fed animals. Insulin injection caused a decrease of glucose levels with respect to basal glucose values by about 30% in group A, group B, and group D at 15, 30, and 60 minutes, whereas the decrease of blood glucose in group C was significantly less. Significance: $^*P < .05$ versus group A. n = 3–4.

are few reports showing the effects of NO-1886 on glucose metabolism. Hara and coworkers [15] demonstrated an effect of enhancing whole-body insulin sensitivity of NO-1886 supplementation. Kusunoki and colleagues [12] reported that NO-1886 suppressed accumulation of visceral fat and reduced insulin resistance in high fat (safflower oil) feeding–induced obesity type 2 diabetes animal model rats evaluated by euglycemic hyperinsulinemic clamp [11]. It needs to be noted that the above studies were generally short-term investigations and there were no intensive observations on the changes of glucose levels. Recently, we used our own high fat/high sucrose feeding–induced type 2 diabetes rabbits model [6] to ascertain the effects of long-term administration of NO-1886 on atherosclerosis. By chance, we had an unexpected finding that NO-1886 reduced blood glucose level in high fat/high sucrose–fed rabbits [7]. However, the mechanism of plasma glucose reduction by NO-1886 cannot be clearly explained from the results of our previous study without performing the glucose tolerance test or insulin tolerance test in our rabbit model.

In the current study, we evaluated whole-body insulin sensitivity using IVGTT and insulin tolerance test, and revealed a significant impact of NO-1886 supplementation on glucose clearance and acute insulin response to intravenous glucose loading in our mild diabetes rabbit model. This would indicate a meaningful role of NO-1886 in suppressing postprandial blood glucose levels. Our results showed that in the fasting state, the increase in glucose levels by feeding the high-fat/high-sucrose diet was inhibited by supplementing 1% NO-1886 into the high-fat/high-sucrose diet at the 8th week time point (4 weeks after start of NO-1886 administration); fasting blood glucose levels in NO-1886–supplemented high fat/high sucrose–fed rabbits were significantly lower than in rabbits that were fed solely the high-fat/high-sucrose diet at the 23rd and 28th weeks (Figure 5). However, we did not obtain a significant effect of NO-1886 on fasting insulin, so that the decrease of fasting glucose would be due to improved insulin sensitivity through the actions of NO-1886 on plasma triglycerides and FFAs as shown in this study.

NO-1886 was primarily developed as a hypolipidemic agent [8, 9]. It has been reported to increase lipoprotein lipase enzyme mass in postheparin plasma, causing a reduction in plasma TG level, with concomitant elevation of HDL-C, by increasing lipoprotein lipase activity in normal and diabetic rats. Diabetes is associated with progressive development of dyslipidemia, which is a risk factor for cardiovascular disease and arteriosclerosis, as well as a cause for lipotoxicity to the liver, muscles, and pancreatic islets [1–3, 15–18]. It has been well known that significant elevations in plasma TG and VLDL-C, and significant reduction in HDL-C, appear to be characteristic in diabetic patients. However, present treatment of diabetes by low-energy diets, oral hypoglycemic agents, and insulin therapy frequently is not sufficient for perfect control of lipid metabolism [16–19]. A drug, such as NO-1886, that has a potential effect on dyslipidemia and beneficial actions in glucose metabolism, as we demonstrated, would have great implication in the treatment of diabetes and deserves further studies. The high-fat/high-sucrose diet would have induced lipotoxicity to the animals' liver, muscles, and pancreatic islets that could have important influence on glucose metabolism. The known actions of NO-1886 on lipoprotein lipase, plasma TG level, and abdominal fat accumulation might account for the effects that we reported in the current study, such as lowering blood glucose level, protecting the acute insulin response to the glucose loading, and improving the animal's insulin sensitivity.

In summary, the results of the current study suggest that NO-1886 suppress the elevation of blood glucose in rabbits induced by feeding a high-fat/high-sucrose diet probably through controlling lipid metabolism and improving insulin sensitivity.

### REFERENCES

[1] Sivitz, W. I. (2001) Lipotoxicity and glucotoxicity in type 2 diabetes. Effects on development and progression. *Postgrad. Med.,* **109,** 55–59.
[2] Groop, L. (2000) Pathogenesis of type 2 diabetes: The relative contribution of insulin resistance and impaired insulin secretion. *Int. J. Clin. Pract. Suppl.,* **113,** 3–13.
[3] Leibowitz, G., Yuli, M., Donath, M. Y., et al. (2001) Beta-cell glucotoxicity in the *Psammomys obesus* model of type 2 diabetes. *Diabetes,* **50(Suppl 1),** S113–S117.
[4] Jörns, A., Tiedge, M., Ziv, E., Shafrir, E., and Lenzen, S. (2002) Gradual loss of pancreatic beta-cell insulin, glucokinase and GLUT2 glucose transporter immunoreactivities during the time course of nutritionally induced type-2 diabetes in *Psammomys obesus* (sand rat). *Virchows Arch.,* **440,** 63–69.
[5] Porte, D. (1999) Mechanisms for hyperglycemia in the metabolic syndrome: The key role of $\beta$-cell dysfunction. *Ann. N.Y. Acad. Sci.,* **892,** 73–83.
[6] Thresher, J. S., Podolin, D. A., Wei, Y., Mazzeo, R. S., Pagliassotti, M. J. (2000) Comparison of the effects of sucrose and fructose on insulin action and glucose tolerance. *Am. J. Physiol. Regul. Integr. Comp. Physiol.,* **279,** R1334–R1340.
[7] Yin, W., Tsutsumi, K., Yuan, Z., and Yang, B. (2002) A lipoprotein lipase activator NO-1886 suppresses atherosclerosis in aorta of mild diabetic rabbits. *Arzneimittel. Forschung. Drug Res.,* **52,** 610–614.
[8] Yin, W., Yuan, Z., and Yang, B. (2002) A diet high in saturated fat and sucrose alters glucoregulation, and induces aortic fatty streaks in New Zealand White rabbits. *Int. J. Exp. Diabetes Res.,* **3,** 179–184.
[9] Tsutsumi, K., Inoue, Y., Shima, A., et al. (1993) The novel compound NO-1886 increases lipoprotein lipase activity with resulting elevation of high density lipoprotein cholesterol, and long-term administration inhibits atherogenesis in the coronary arteries of rats with experimental atherosclerosis. *J. Clin. Invest.,* **92,** 411–417.
[10] Chiba, T., Miura, S., Sawamura, F., et al. (1997) Antiatherogenic effects of a novel lipoprotein lipase-enhancing agent in cholesterol-fed New Zealand White rabbits. *Arterioscler. Thromb. Vasc. Biol.,* **17,** 2601–2608.
[11] Hara, T., Kusunoki, M., Tsutsumi, K., et al. (1998) A lipoprotein lipase activator, NO-1886, improves endothelium-dependent relaxation of rat aorta associated with aging. *Eur. J. Pharmacol.,* **350,** 75–79.
[12] Kusunoki, M., Hara, T., Tsutsumi, K., et al. (2000) The lipoprotein lipase activator, NO-1886, suppresses fat accumulation and insulin resistance in rats fed a high-fat diet. *Diabetologia,* **43,** 875–880.
[13] Tsutsumi, K., Inoue, Y., Shima, A., et al. (1995) Correction of hypertriglyceridemia with low high-density lipoprotein cholesterol by the novel compound NO-1886, a lipoprotein lipase-promoting agent, in STZ-induced diabetic rats. *Diabetes,* **44,** 414–417.
[14] Tsutsumi, K., Inoue, Y., and Murase, T. (2000) Effects of NO-1886, a lipoprotein lipase promoting agent on homozygous and heterozygous WHHL rabbits. *Arzneim. Forsch.,* **50,** 118–121.
[15] Hara, T., Cameron-Smith, D., Cooney, G. J., et al. (1998) The action of novel lipoprotein lipase activator, NO-1886, in hypertriglyceridemic fructose-fed rats. *Metabolism,* **47,** 149–153.
[16] Ginsberg, H. N. (1996) Diabetic dyslipidemia: Basic mechanisms underlying the common hypertriglyceridemia and low HDL cholesterol levels. *Diabetes,* **45(Suppl 3),** S27-S30.
[17] Garg, A. (1998) Treatment of diabetic dyslipidemia. *Am. J. Cardiol.,* **81,** 47B–51B.
[18] Taskinen, M. R. (2001) Pathogenesis of dyslipidemia in type 2 diabetes. *Exp. Clin. Endocrinol. Diabetes,* **109(Suppl 2),** S180–S188.
[19] Taskinen, M. R. (2002) Diabetic dyslipidemia. *Atheroscler. Suppl.,* **3,** 47–51.

